# Supplementary material for: Investigation of anti-proliferative and anti-angiogenic properties of Parkia javanica bark and fruit extracts in zebrafish
Source: PLoS One. 2023 Jul 21;18(7):e0289117. doi: 10.1371/journal.pone.0289117 (PMC10361473; doi:10.1371/journal.pone.0289117)
Supplement: S1 Table — (DOCX) [file pone.0289117.s001.docx]

**Supplementary Table 1: Taxonomic identification of *Parkia javanica***

**Parkia javanica (Lam.) Merr.**

Bentham and Hooker (1862-1883)

| Division | Polypetally |
| --- | --- |
| Series | Calyciflorae |
| Cohort: | Rosales |
| Order: | Leguminasae |
| Genus: | *Parkia* |
| Species: | **j***avanica* |

# Authentication:

# The authentication of the plant was done by Dr. H. J. Chowdhary, the Joint Director, Central National Herbarium, Botanical Survey of India, Shibpur, Howrah, West Bengal. The voucher specimen No. ≠ BD-01/06 has been deposited in the Herbarium.
