# Supplementary material for: Investigation of anti-proliferative and anti-angiogenic properties of Parkia javanica bark and fruit extracts in zebrafish
Source: PLoS One. 2023 Jul 21;18(7):e0289117. doi: 10.1371/journal.pone.0289117 (PMC10361473; doi:10.1371/journal.pone.0289117)
Supplement: S2 Table — (DOCX) [file pone.0289117.s002.docx]

**Supplementary Table 2: GC-MS library of *Parkia javanica* fruit extract**

| **Sl.no.** | **Compound Name** | **Area %** | **RT(min)** | **M.W** | **CAS** | **Formula** |
| --- | --- | --- | --- | --- | --- | --- |
| 1 | Phenol, 2,4-bis(1,1-dimethylethyl) | 1.101 | 24.964 | 206 | 96-76-4 | C14H22O |
| 2 | Heptadecanoic acid, heptadecyl ester | 1.796 | 35.033 | 508 | 36617-50-2 | C34H68O2 |
| 3 | Humulane-1,6-dien-3-ol | 1.273 | 38.505 | 222 | 900140-23-1 | C15H26O |
| 4 | 17-pentatriacontene | 1.166 | 38.615 | 490 | 6971-40-0 | C35H70 |
| 5 | Globulol | 1.295 | 38.705 | 222 | 51371-47-2 | C15H26O |
| 6 | Gamma.-sitosterol | 3.157 | 39.640 | 414 | 83-47-6 | C29H50O |
| 7 | 28-norolean-17-en-3-one | 3.685 | 42.697 | 410 | 5912-72-1 | C29H46O |
| 8 | Lupeol | 6.377 | 43.492 | 426 | 545-47-1 | C30H50O |
| 9 | Dihydroartemisinin, 10-o-(t-butyloxy) | 1.659 | 43.697 | 356 | 139727-23-4 | C19H32O6 |
| 10 | 1-cyclohexene-1-acrylic acid, 2,6,6-trimethyl-3-oxo | 24.015 | 44.382 | 222 | 28043-03-0 | C13H18O3 |
| 11 | Lup-20(29)-en-3-ol, acetate, (3.beta.) | 12.852 | 44.982 | 468 | 1617-68-1 | C32H52O2 |
| 12 | Stigmast-4-en-3-one | 2.249 | 46.458 | 412 | 1058-61-3 | C29H48O |
| 13 | 3-isopropoxy-1,1,1,5,5,5-hexamethyl-3-(trimethylsilox) | 1.219 | 49.414 | 354 | 72182-11-7 | C12H34O4Si4 |
| 14 | 1,2-bis(trimethylsilyl)benzene | 1.282 | 53.071 | 222 | 17151-09-6 | C12H22Si2 |
