# Supplementary material for: Investigation of anti-proliferative and anti-angiogenic properties of Parkia javanica bark and fruit extracts in zebrafish
Source: PLoS One. 2023 Jul 21;18(7):e0289117. doi: 10.1371/journal.pone.0289117 (PMC10361473; doi:10.1371/journal.pone.0289117)
Supplement: S3 Table — (DOCX) [file pone.0289117.s003.docx]

**Supplementary Table 3: GC-MS library of *Parkia javanica* bark extract**

| Compound  No | **m/z ratio** | Molecular  weight | **Molecular formula** | **Area %** | **Identified Chemical Compounds in MEPJB** |
| --- | --- | --- | --- | --- | --- |
| C: 1 | 191.1774 | 206 | C_14_H_22_O | 50.740% | Phenol,2,4-bis(1,1-dimethylethyl)- OR 2,4-Di-tert-butylphenol (2,4-DTBP) |
| C: 2 | 73.9704 | 340 | C_22_H_44_O_2_ | 2.552% | Methyl 14-methyl-eicosanoate |
| C: 3 | 149.0541; 72.8706 | 652 | C_38_H_68_O_8_ | 4.023% | L-(+)-ascorbic acid 2,6-dihexadecanoate |
| C: 4 | 56.9816 | 276 | C_17_H_24_O_3_ | 3.387% | 7,9-di-tert-butyl-1-oxaspiro(4,5)deca-6,9-diene-2,8-dione |
| C: 5 | 149.1226 | 278 | C_16_H_22_O_4_ | 6.636% | Dibutyl phthalate (DBP) |
| C: 6 | 73.9704 | 256 | C_16_H_32_O_2_ | 3.300% | Methyl 9-methyltetradecanoate |
| C: 7 | 72.9394 | 256 | C_16_H_32_O_2_ | 5.716% | n-Hexadecanoic acid |
| C: 8 | 149.0541 | 352 | C_23_H_44_O_2_ | 5.281% | Methyl 11-Docosenoate |
| C: 9 | 56.9128 | 436 | C_31_H_64_ | 4.171% | Hentriacontane |
| C: 10 | 56.9128 | 914 | C_54_H_108_Br_2_ | 10.106% | Tetrapentacontane, 1,54-dibromo- |
| C: 11 | 56.9128 | 696 | C_41_H_77_O_2_F_5_ | 4.900% | Octatriacontyl pentafluoropropionate |
| C: 12 | 59.8720 | 180 | C_6_H_12_O_6_ | 20.870% | D-Allose |
| C: 13 | 73.9017 | 228 | C_14_H_28_O_2_ | 3.590% | Methyl 11-Methyl-Dodecanoate |
| C: 14 | 54.9166 | 296 | C_19_H_36_O_2_ | 4.066% | Methyl 13-Octadecenoate |
| C: 15 | 56.9816 | 322 | C_23_H_46_ | 2.352% | 5-Methyl-Z-5-Docosene |
