# Supplementary material for: Investigation of anti-proliferative and anti-angiogenic properties of Parkia javanica bark and fruit extracts in zebrafish
Source: PLoS One. 2023 Jul 21;18(7):e0289117. doi: 10.1371/journal.pone.0289117 (PMC10361473; doi:10.1371/journal.pone.0289117)
Supplement: S1 File — (DOCX) [file pone.0289117.s004.docx]

**Highlights:**

- Secondary metabolites of *Parkia javanica* exhibit diverse pharmacological activities.
- The Danio rerio (Zebrafish) is an optimistic in vivo model in biomedical research for its dynamics and in understanding the aetiology of diseases.
- *Parkia javanica* extracts showed toxicity with alteration in neovascularisation in the zebrafish model system.
- The pro-apoptotic and anti-proliferative role of *Parkia javanica* extracts will unlock new approaches in cancer therapeutics.
- Combination of traditional diet and modern phytochemical medicine can be a holistic approach to treating and managing cancer.
