# Supplementary material for: Investigation of anti-proliferative and anti-angiogenic properties of Parkia javanica bark and fruit extracts in zebrafish
Source: PLoS One. 2023 Jul 21;18(7):e0289117. doi: 10.1371/journal.pone.0289117 (PMC10361473; doi:10.1371/journal.pone.0289117)
Supplement: Attachment — Submitted filename: Response to Reviewers comments.docx [file pone.0289117.s005.docx]

**Response to reviewer’s comments**

**Journal name:** PLOS ONE

**Manuscript ID: PONE-D-22-33455**

**Title:** Investigation of anti-proliferative and anti-angiogenic properties of *Parkia javanica* bark and fruit extracts in zebrafish

**All the modifications in the revised manuscript are highlighted in yellow colour.**

**Comments to the Author**
1. Is the manuscript technically sound, and do the data support the conclusions?
The manuscript must describe a technically sound piece of scientific research with data that supports the conclusions. Experiments must have been conducted rigorously, with appropriate controls, replication, and sample sizes. The conclusions must be drawn appropriately based on the data presented.

Reviewer #1: Partly

Reviewer #2: Partly

2. Has the statistical analysis been performed appropriately and rigorously?

 Reviewer #1: No

Reviewer #2: No

3. Have the authors made all data underlying the findings in their manuscript fully available?
The [PLOS Data policy](http://www.plosone.org/static/policies.action#sharing) requires authors to make all data underlying the findings described in their manuscript fully available without restriction, with rare exception (please refer to the Data Availability Statement in the manuscript PDF file). The data should be provided as part of the manuscript or its supporting information, or deposited to a public repository. For example, in addition to summary statistics, the data points behind means, medians and variance measures should be available. If there are restrictions on publicly sharing data—e.g. participant privacy or use of data from a third party—those must be specified.

Reviewer #1: Yes

Reviewer #2: No

4. Is the manuscript presented in an intelligible fashion and written in standard English?
PLOS ONE does not copyedit accepted manuscripts, so the language in submitted articles must be clear, correct, and unambiguous. Any typographical or grammatical errors should be corrected at revision, so please note any specific errors here.

Reviewer #1: Yes

Reviewer #2: No

5. Review Comments to the Author
Please use the space provided to explain your answers to the questions above. You may also include additional comments for the author, including concerns about dual publication, research ethics, or publication ethics. (Please upload your review as an attachment if it exceeds 20,000 characters)

Reviewer #1:

**In the manuscript “Investigation of anti-proliferative and anti-angiogenic properties of Parkia javanica bark and fruit extracts in zebrafish” authors have compared the anti-proliferative and anti-angiogenic properties of bark and fruit extracts of Parkia javanica in the zebrafish model. In addition, authors have reported the efficacy of fruit extract in inhibiting the proliferation of cells compared to bark extract. Although the results are interesting, it is a preliminary study and requires extensive studies in appropriate model systems to demonstrate the anti-proliferative and anti-angiogenic properties. Furthermore, as authors have also mentioned, identification of active ingredients is lacking in this manuscript. At least authors would have determined the composition of the extract by LC-MS-MS. In light of these shortcomings, I recommend the publication of this manuscript only after including the composition analysis of the extract and addressing the following comments**

***Response:*** Thank you for your valuable suggestion. The comments are well taken. We have carried out GC-MS analysis of the extracts to understand the composition of the crude extracts obtained from the fruit and the bark. However, in this manuscript the focus was on analysing the effects of the crude extracts. In the revised manuscript, the composition analysis of the crude extracts have been mentioned briefly in the discussion section (Lines 264-271) and the details are included as supplemental data (Table 2 and 3).

Major comments

**1. Did a certified botanist authenticate the plant? If so provide the authentication certificate and the photograph of the specimen used for authentication as supplemental data. Mention the full name of the plant Parkia javanica (Lam.) Merr.**

***Response:*** We thank the reviewer for this suggestion. Yes, the taxonomic identification of the plant was done by a certified botanist , Dr H.J. Chowdhary, the joint director at the Central National Herbarium, Botanical Survey of India, Shibpur, Howrah, West Bengal, India. As per the suggestion of the reviewer, the details of this identification are shown as supplemental table 1.

**2. Did authors use whole fruit for the extract preparation? Or they have separated the skin and seeds?**

***Response:*** We thank the reviewer for this comment. Yes, the whole fruit, without separating the skin and the seeds was used for the crude extract preparation.

**3. How much is the yield of methanolic extract?**

***Response:*** Thank you for your valuable suggestion. The percentage yield is now mentioned in the materials section in the revised manuscript. (Line 110)

**4. Error bars are missing in all the bar graphs. Did authors determine the statistical significance? If so, add the information**

***Response:*** We thank the reviewer for this valuable suggestion. As per the suggestions, the error bars are included in the bar graphs for all the figures.

Minor comments
**Line #94. Authors have mentioned that they have prepared extracts from bark as well as fruit, but the name MEPJB, referring extract from bark, was only mentioned. Did authors have labelled the extract fruit as MEPJF? If so, include it in the methods section.**

***Response:*** Thank you for your valuable suggestion. Corrections have been made in the revised manuscript according to the reviewer’s suggestion (Lines 105-106).

**Line #129: Mention the concentration of polythiourea**

***Response:*** Thank you for your valuable suggestion. Corrections have been made in the revised manuscript according to reviewer’s suggestion the concentration of PTU is indicated in the method section (Line 141).

**Reviewer #2:**

**[1]. The abstract does not provide much context for why the study is important or what is already known about the topic. It would be helpful to include a brief introduction that explains the prevalence and anti-proliferative and anti-angiogenic properties, and why finding new treatments is important.**

***Response:*** We thank the reviewer for this suggestion. We have now added a few sentences highlighting the relevance of the study (Lines 23-26). However, since the abstract has certain word limits, we couldn’t add to many information there. However, explanations on why new treatments are important are mentioned in the introduction part (Lines 72-82).

**[2]. The abstract does not provide information on the sample size and experimental design of the study, which is an important factor in determining the reliability of the results. Providing more information on these aspects would help readers to understand the study's rigor.**

***Response:*** We thank the reviewer for this suggestion. For a study of this nature, sample size is not very informative as the focus was on determining the anti-cancer properties of crude extracts of fruit and bark of a plant, *Parkia javanica*. However, as per the suggestion, we have now included a few sentences about the experimental design in the abstract of the revised manuscript (Lines 31-33).

**[3]. In line 52, the Manipuri name of the plant is wrong.**

***Response:*** Thank you for your valuable suggestion. Corrections in the local name of the plant have been made in the revised manuscript according to reviewer’s suggestion (Line 56) **[4]. What about the authentication of plant material?**

***Response:*** Thank you for your valuable suggestion. The details of authentication of the plant material have been added in the revised manuscript according to reviewer’s suggestion as a supplemental data and the same has been mentioned in the methods section. (Lines 101-102).  **[5]. Voucher specimen no is missing in the MS.**

***Response:*** Thank you for your valuable suggestion. The details of voucher specimen no has been added as a supplemental data in the revised manuscript.

**[6]. A list of chemical and reagent are missing.**

***Response:*** Thank you for your valuable suggestion. The list of chemical and reagents used are incorporated in the materials and methods section in the revised manuscript (Lines 175-179).

**[7]. While using the rotary evaporator, the temperature should not be more than 45-48 oC but in the current work 60 ^o^C was used which is not acceptable.**

***Response:*** Thank you for your valuable correction. The temperature for the rotary evaporator was fixed at 45**^o^**C. It was a typographical error, it has been modified in the revised manuscript.  **[8]. As mentioned in lines 97-99 “Finally, the concentrated solution was lyophilized at 4°C to eliminate the methanol..”, but lyophilization is important for removing the water traces, not the methanol.**

***Response:*** Thank you for your valuable correction. We have not lyophilized the samples. It was simply dried at 4C for completely removal of methanol. The word lyophilized have been replaced with dried in the revised manuscript (line 109). **[9]. The % yield of the extract is missing.**

***Response:*** Thank you for your valuable comment. The yield of the extract has been incorporated in the revised method section (Line 110).

**[10]. There is no details section of statistical analysis.**

The details of statistical significance is mentioned in the materials and methods section (Line 181-183).
**[11]. The authors did not clarify the standard drug used in the study.**

We would like to clarify here that there was no standard drug used in this study. The observations were compared between the embryos treated with crude extracts and those untreated.
**[12]. It would be good if the phyto constituent of the active extract can be detected by HPLC or LC-MS or some other analytical techniques.**

***Response:*** Thank you for your valuable comment. The GC-MS composition of both the fruit and the bark extract are shown as supplementary data (Supplementary tables 2 and 3) in the revised manuscript.

**[13]. The presentation of the results and discussion is not well organized.**

***Response:*** Thank you for your valuable comment. In the revised manuscript, the sections pertaining to the presentation of the results and the discussion were rephrased for better clarity.
**[14]. The conclusion is not effective.**

***Response:*** Thank you for your valuable comment. The conclusion part has been rephrased.

**[15]. There are many syntax errors, confusing sentences, and grammatical mistakes in the manuscript. The manuscript critically needs revision.**

***Response:*** Thank you for your valuable comment. As per the reviewer’s suggestion, the entire manuscript has been revised extensively to remove the syntax errors and grammatical errors.

**[16]. The English of the manuscript is not up to the standard of the journal.**

***Response:*** Thank you for your valuable comment. The manuscript has been edited extensively to ensure that the language used is as scientific as possible and the usage of the vocabulary is as per the requirements of scientific journals.

6. PLOS authors have the option to publish the peer review history of their article ([what does this mean?](https://journals.plos.org/plosone/s/editorial-and-peer-review-process#loc-peer-review-history)). If published, this will include your full peer review and any attached files.


If you choose “no”, your identity will remain anonymous but your review may still be made public.


**Do you want your identity to be public for this peer review?** For information about this choice, including consent withdrawal, please see our [Privacy Policy](https://www.plos.org/privacy-policy).

 Reviewer #1: **Yes: SubbaRao V. Madhunapantula**

Reviewer #2: No

[NOTE: If reviewer comments were submitted as an attachment file, they will be attached to this email and accessible via the submission site. Please log into your account, locate the manuscript record, and check for the action link "View Attachments". If this link does not appear, there are no attachment files.]

While revising your submission, please upload your figure files to the Preflight Analysis and Conversion Engine (PACE) digital diagnostic tool, <https://pacev2.apexcovantage.com/>. PACE helps ensure that figures meet PLOS requirements. To use PACE, you must first register as a user. Registration is free. Then, login and navigate to the UPLOAD tab, where you will find detailed instructions on how to use the tool. If you encounter any issues or have any questions when using PACE, please email PLOS at [figures@plos.org](mailto:figures@plos.org). Please note that Supporting Information files do not need this step.
